# The effectiveness of high dose zinc acetate lozenges on various common cold symptoms: a meta-analysis

**DOI:** 10.1186/s12875-015-0237-6

**Published:** 2015-02-25

**Authors:** Harri Hemilä, Elizabeth Chalker

**Affiliations:** Department of Public Health, POB 41, University of Helsinki, Mannerheimintie 172, FIN-00014 Helsinki, Finland; University of Sydney, Sydney, Australia

**Keywords:** Common cold, Cough, Laryngitis, Meta-analysis, Myalgia, Randomized controlled trials, Pharyngitis, Respiratory tract infections, Rhinitis, Zinc acetate

## Abstract

**Background:**

A previous meta-analysis found that high dose zinc acetate lozenges reduced the duration of common colds by 42%, whereas low zinc doses had no effect. Lozenges are dissolved in the pharyngeal region, thus there might be some difference in the effect of zinc lozenges on the duration of respiratory symptoms in the pharyngeal region compared with the nasal region. The objective of this study was to determine whether zinc acetate lozenges have different effects on the duration of common cold symptoms originating from different anatomical regions.

**Methods:**

We analyzed three randomized trials on zinc acetate lozenges for the common cold administering zinc in doses of 80–92 mg/day. All three trials reported the effect of zinc on seven respiratory symptoms, and three systemic symptoms. We pooled the effects of zinc lozenges for each symptom and calculated point estimates and 95% confidence intervals (95% CI).

**Results:**

Zinc acetate lozenges shortened the duration of nasal discharge by 34% (95% CI: 17% to 51%), nasal congestion by 37% (15% to 58%), sneezing by 22% (−1% to 45%), scratchy throat by 33% (8% to 59%), sore throat by 18% (−10% to 46%), hoarseness by 43% (3% to 83%), and cough by 46% (28% to 64%). Zinc lozenges shortened the duration of muscle ache by 54% (18% to 89%), but there was no difference in the duration of headache and fever.

**Conclusions:**

The effect of zinc acetate lozenges on cold symptoms may be associated with the local availability of zinc from the lozenges, with the levels being highest in the pharyngeal region. However our findings indicate that the effects of zinc ions are not limited to the pharyngeal region. There is no indication that the effect of zinc lozenges on nasal symptoms is less than the effect on the symptoms of the pharyngeal region, which is more exposed to released zinc ions.

Given that the adverse effects of zinc in the three trials were minor, zinc acetate lozenges releasing zinc ions at doses of about 80 mg/day may be a useful treatment for the common cold, started within 24 hours, for a time period of less than two weeks.

**Electronic supplementary material:**

The online version of this article (doi:10.1186/s12875-015-0237-6) contains supplementary material, which is available to authorized users.

## Background

Interest in zinc lozenges (as tablets that are intended to be dissolved slowly in the mouth) for the treatment of the common cold started from the serendipitous observation that the cold symptoms of a 3-year-old girl with leukemia disappeared within a few hours when she slowly dissolved a therapeutic zinc tablet in her mouth instead of swallowing it [1]. The benefit appeared to be derived from dissolving the tablet in the mouth, which implied that zinc may have local effects in the pharyngeal region. This observation led the father of the child to conduct a randomized trial, which found that zinc lozenges significantly shortened colds [1].

Subsequently a series of zinc lozenge trials were carried out but with variable results. The composition of the zinc lozenges differed between trials with some lozenges containing substances that tightly bind to zinc ions, such as citric acid. Therefore, variation in the level of free zinc ions has been proposed as one factor that can explain the significant heterogeneity in the results of the trials [2-10]. Acetate does not chemically bind to zinc ions and therefore zinc acetate may be an ideal zinc salt for composing lozenges that release high levels of free zinc ions [6,9,10].

A recent meta-analysis investigated the role of zinc dosage on the effect of zinc lozenges on cold duration [11]. Five trials with zinc lozenges that contained low doses of zinc, <75 mg/day, consistently found no effect from the lozenges. In contrast, three trials with high doses of zinc, >75 mg/day, as zinc acetate lozenges, consistently found that colds were shortened by a mean of 42% [11]. Five high-dose trials used zinc salts other than acetate and obtained a mean 20% reduction in cold duration [11]. Two other systematic reviews on zinc and the common cold combined zinc lozenge trials with zinc syrup trials [12,13]. Those reviews however overlooked the fact that slowly dissolving zinc lozenges may cause local effects in the pharyngeal region, whereas rapidly swallowed syrup does not. In addition, the two reviews had other severe limitations [14-16].

Nasal administration of zinc shortened the duration of colds in two studies [17,18], which implied that zinc can have a local effect against colds within the nasal region. Similarly, the effects of zinc lozenges may be local within the pharyngeal region instead of being purely systemic. Although the local effects of zinc in the nasal region are of interest for the purposes of researching the mechanisms of the effects of zinc, nasal zinc application might cause anosmia [19,20] and nasal zinc application should be discouraged unless application methods are developed that eliminate such a risk.

When zinc acetate lozenges dissolve in the mouth, zinc ions are released into the saliva of the pharyngeal region where the levels are consequently high. However, zinc lozenges do not seem to increase the level of zinc in the nasal mucosa ([6], p490). If the zinc ion concentration of the mucosa determines the effect of zinc lozenges on the specific symptoms on the particular anatomical region, there might be substantial differences between the effects of zinc on throat symptoms compared with nasal symptoms.

The purpose of this meta-analysis is to investigate whether zinc acetate lozenges have different effects on the duration of common cold symptoms originating from different anatomical regions.

## Methods

### Selection of the trials

This meta-analysis was restricted to placebo-controlled trials on the effect of zinc acetate lozenges on natural common cold infections in which the doses were >75 mg/day of zinc ion. Previous searches of the literature [11-13] identified three trials that fulfilled the inclusion criteria [21-23] (Additional file 1). Table 1 summarises the characteristics of the three included trials. No additional zinc acetate trials were found by searching PubMed using the free search terms “zinc” and “lozenge$” (Jan 2, 2015). This was an analysis of published data and therefore no ethics approval was needed. This study is reported according to the PRISMA Statement [24].

**Table 1:** **Characteristics of high dose zinc acetate lozenge trials**

**Trial**	**Characteristics**
**Petrus et al. (1998) [** 21 ]*	
Methods	Randomized, placebo-controlled, double-blind trial.
Participants	Included in the analysis: 52 Zn and 49 placebo participants: 47 M 54 F, mean age 26 yr (range 18 to 54 yr). Participants were recruited from the campus of the University of Texas through posted announcements. Exclusions: serious illnesses, organ transplants, disability.
Intervention	Zn acetate: one lozenge contained 9 mg Zn. Placebo lozenges contained sucrose octaacetate. Participants were instructed to use 1 lozenge every 1½ hr while awake during day 0, then 1 lozenge every 2 hr while awake on following days. The mean number of lozenges used per day by all participants was 9.9. Mean daily zinc dose was 89 mg/d. 97 of the 101 subjects started using zinc lozenges on the first day of enrollment in the study (4 on day 2), but data on the length of time between onset of symptoms and enrollment is not available.
Common cold definition	Presence of ≥2 of the following symptoms: nasal drainage, nasal congestion, cough, fever, myalgia, headache, sore throat, scratchy throat, hoarseness, sneezing, malaise.
**Prasad et al. (2000) [** 22 **]**	
Methods	Randomized, placebo-controlled, double-blind trial.
Participants	Included in the analysis: 25 Zn and 23 placebo participants: 18 M 30 F, mean age 37 yr (SD 11 yr). Participants were students, staff, and employees at Wayne State University, Michigan, who were ≥18 yr. Exclusions: pregnancy, a known immunodeficiency disorder, chronic illnesses, previous use of zinc lozenges. Inclusion required that the cold had lasted for ≤24 hr.
Intervention	Zn acetate: one lozenge contained 12.8 mg Zn. Placebo lozenges contained sucrose octaacetate. Participants were instructed to dissolve 1 lozenge in their mouth every 2 to 3 hr while awake. The mean number of lozenges used per day in the Zn group was 6.2. Mean daily zinc dose was 80 mg/d.
Common cold definition	Presence of ≥2 of the following symptoms: cough, headache, hoarseness, muscle ache, nasal discharge, nasal congestion, scratchy throat, sore throat, sneezing, fever.
**Prasad et al. (2008) [** 23 **]**	
Methods	Randomized, placebo-controlled, double-blind trial
Participants	Included in the analysis: 25 Zn and 25 placebo participants: 16 M 34 F, mean age 35 yr (SD 14 yr). Participants were students, staff, and employees at Wayne State University, Michigan, who were ≥18 yr. Exclusions: pregnancy, any known immune deficiency disorder or chronic illness, previous use of zinc lozenges. Inclusion required that the cold had lasted for ≤24 hr.
Intervention	Zn acetate: one lozenge contained 13.3 mg Zn. Placebo lozenges contained sucrose octaacetate. The packages were identical in appearance except for the randomization numbers. Participants were asked to dissolve 1 lozenge in their mouth every 2 to 3 hr while awake. The mean number of lozenges used per day in the Zn group was 6.9. Mean daily zinc dose was 92 mg/d.
Common cold definition	Presence of ≥2 of the following symptoms: cough, headache, hoarseness, muscle ache, nasal discharge, nasal congestion, scratchy throat, sore throat, sneezing, fever.

*Additional information about [21] was received from Kenneth Lawson on Jan 7, 2015.

### Outcomes and extraction of data

Each of the three trials reported the total duration of colds and the duration of the same respiratory and systemic symptoms. The extracted original data are available in Additional file 2. Petrus et al. [21] reported the results to only one decimal place; more precise data were kindly provided by Kenneth Lawson for the total duration of colds on March 4, 2009 and for the duration of specific symptoms of colds on April 11, 2014. Both authors of this study checked the accuracy of the extracted data against the original reports and by referring directly to Lawson’s emails.

### Statistical methods

Differences in the distributions of viruses and in the severity of disease in different patient groups in addition to differences in outcome definitions cause considerable variation in the recorded durations of colds in different untreated patient groups. Therefore, the relative effect of zinc on the common cold duration was calculated in percentages, because the relative effect adjusts for variations between the patient groups and outcome definitions.

In the original study reports, the duration of each symptom was reported in days. For this meta-analysis, the durations were transformed to a percentage scale so that the duration of each symptom in the corresponding placebo group was 100%. Consequently the pooling of the three studies gives the percentage effect of zinc acetate on the specific symptoms. The transformation to the percentage scale is described in Additional file 2.

We pooled the effect of zinc acetate lozenges on the specific symptoms by using the inverse-variance fixed-effect option in the RevMan program [25] (Additional file 3). Heterogeneity between the three studies was assessed by using the χ^2^-test and the I^2^-statistic [26]. The I^2^-statistic estimates the percentage of total variation across studies that is due to true heterogeneity rather than due to chance. A value of I^2^ greater than about 75% indicates a high level of heterogeneity. Some of the RevMan forest plots were redrawn with Gnuplot [27] for greater clarity.

To compare the mean durations of the specific symptoms in the placebo groups of each trial, we transformed the durations of the specific symptoms to a percentage of the total mean common cold duration for each group (Additional file 2). Thereafter the durations of the specific symptoms for all three trials were pooled using the RevMan program (Additional file 4). This pooling gave the duration of the specific untreated symptom as a percentage of the total untreated common cold duration. Both authors checked the accuracy of the calculations.

## Results

Three studies were found which used zinc acetate lozenges containing zinc in doses of over 75 mg/day. The trials were randomized, placebo-controlled and double-blind, and few drop-outs occurred in the trials (Tables 1 and 2). The dose of zinc varied from 80 to 92 mg/day in the three studies. In total, there were 102 participants in the zinc groups and 97 participants in the placebo groups.

**Table 2:** **Methodological characteristics of the included trials**

**Study, Domain of interest**	**Description**
**Petrus et al. 1998 [** 21 ]*	
Randomization	Reported as a randomized trial, but the method of randomization was not described.
Allocation concealment	Participants and personnel did not know to which group the participants were allocated.
Blinding of participants and personnel	Reported as double-blind, which implies that participants and personnel were blinded.
Blinding of outcome assessment	Blinded subjects recorded their symptoms every day.
Losses to follow-up	1 was lost to follow-up.
**Prasad et al. 2000 [** 22 ]*	
Randomization	A Research-Assistant was responsible for randomization. This person did not see the patients and was not involved in collection of clinical data. The subjects were randomized into zinc and placebo groups by the research assistant as they were recruited.
Allocation concealment	Participants and personnel did not know to which group the participants were allocated.
Blinding of participants and personnel	The Clinical Assistant who collected all of the clinical information and remained in touch with the subjects who were recruited for the study remained completely blinded regarding the contents of the zinc and placebo pills.
Blinding of outcome assessment	Blinded participants completed daily logs.
Losses to follow-up	2 in the placebo group dropped out on day 2.
**Prasad et al. 2008 [** 23 ]*	
Randomization	A research consultant prepared the randomization code and the packages of medication. The packages were identical in appearance except for the randomization numbers. This person did not see the patients and was not involved in collection of clinical data. The subjects were randomized into zinc and placebo groups by the research assistant as they were recruited.
Allocation concealment	Participants and personnel did not know to which group the participants were allocated.
Blinding of participants and personnel	The Clinical Assistant who collected all of the clinical information and remained in touch with the subjects who were recruited for the study remained completely blinded regarding the contents of the zinc and placebo pills.
Blinding of outcome assessment	Blinded participants completed daily logs.
Losses to follow-up	No drop outs.

*Additional information about the methods of [22,23] was received from Ananda Prasad on Dec 17, 2014 and of [21] from Kenneth Lawson on Jan 7, 2015.

**Figure 1:**
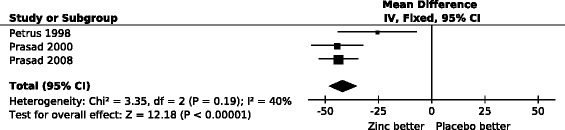
**The effect of high dose zinc acetate lozenges on the duration of the common cold.** In the forest plot on the right side, the vertical line indicates the placebo level. The horizontal lines indicate the 95% CI for the zinc effect and the squares in the middle of the horizontal lines indicate the point estimate of the effect in the particular trial. The sizes of the squares indicate the relative weights of the trials. The diamond shape indicates the pooled effect and the 95% CI. The pooled effect was −42% (95% CI: −35% to −48%; P = 10^−33^). The duration of colds was transformed to the relative scale so that the duration in the respective placebo group was given the value of 100%. Thus the difference between zinc and placebo groups directly indicates the effect of zinc lozenges in percentages. See Additional file 2 for the extraction of data and for the calculation of the relative mean and SD values for total common cold duration.

There was no substantial heterogeneity between the three trials in the effect of zinc acetate lozenges on total common cold duration (P = 0.2; I^2^ = 41%), and a pooled estimate of a 42% reduction in the total duration of the colds was obtained (Figure 1).

**Figure 2:**
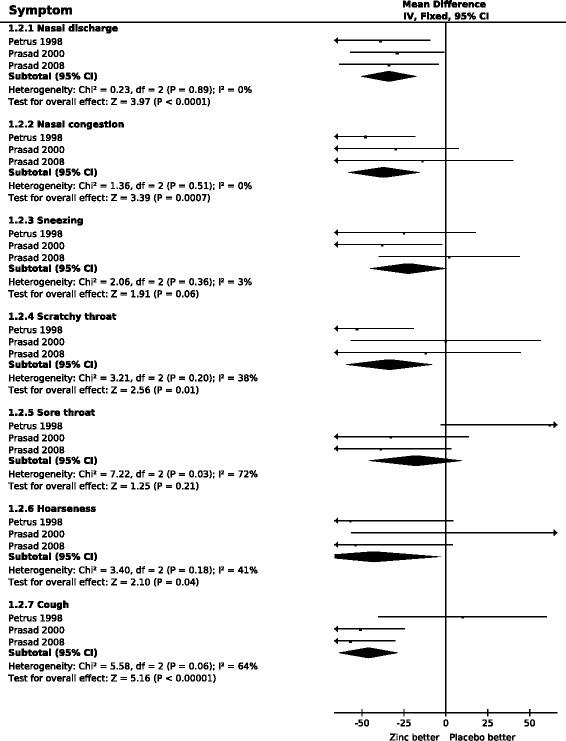
**The effect of high dose zinc acetate lozenges on the duration of respiratory symptoms of the common cold.** In the forest plots on the right side, the vertical line indicates the placebo level. The horizontal lines indicate the 95% CI for the zinc effect and the square in the middle of the horizontal line indicates the point estimate of the effect in the particular trial. Arrows at the end of the horizontal lines indicate that the 95% CI extends out of the forest plot. The sizes of the squares indicate the relative weights of the trials. The diamond shape indicates the pooled effect on the symptoms and its 95% CI. The duration of symptoms was transformed to the relative scale, thus the duration in the respective placebo group was given the value of 100%. The difference between zinc and placebo groups thus directly indicates the effect of zinc lozenges in percentages. See Additional file 2 for the extraction of data and for the calculation of the relative mean and SD values for the duration of symptoms, and Additional file 3 for the raw data and the estimates for individual studies.

In Figure 2, the respiratory symptoms are ordered anatomically so that the three symptoms of the nasal region are at the top of the figure, followed by two throat symptoms, and finally hoarseness (indicating laryngitis) and cough. In the three nasal symptoms, there is no heterogeneity in the zinc acetate lozenge effect between the trials (I^2^ < 3% for each symptom). The pooled estimates indicate that zinc acetate lozenges shorten nasal discharge by 34% and nasal congestion by 37%, whereas the 22% shorter duration of sneezing is not significantly different (Additional file 3).

The zinc lozenge effect on scratchy throat was not substantially heterogeneous between the three trials (I^2^ = 38%); a pooled estimate of a 33% reduction in the duration was calculated (Figure 2). The effect of zinc on sore throat was significantly heterogeneous with I^2^ = 72% (P = 0.03), and the pooled estimate of an 18% shorter duration of sore throat was not significantly different.

The effect of zinc lozenges on hoarseness is not substantially heterogeneous (I^2^ = 41%). None of the three trials individually found a significant effect on hoarseness, but the pooled estimate indicates a 43% decrease in the duration of hoarseness.

Cough was not influenced by zinc in the Petrus et al. study, whereas in the two studies by Prasad et al. a significant reduction in the duration of cough was observed (Figure 2). However, the confidence intervals were wide and heterogeneity was marginally nonsignificant with I^2^ = 64% (P = 0.06). The pooled result indicates a 46% reduction in the duration of cough.

**Figure 3:**
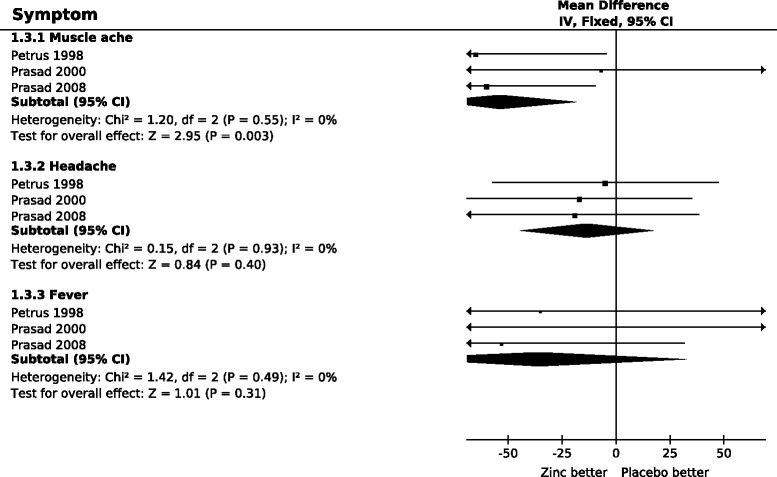
**The effect of high dose zinc acetate lozenges on the duration of systemic symptoms of the common cold.** In the forest plots on the right side, the vertical line indicates the placebo level. The horizontal lines indicate the 95% CI for the zinc effect and the square in the middle of the horizontal line indicates the point estimate of the effect in the particular trial. Arrows at the end of the horizontal lines indicate that the 95% CI extends out of the forest plot. The sizes of the squares indicate the relative weights of the trials. The diamond shape indicates the pooled effect on the symptoms and its 95% CI. The duration of symptoms was transformed to the relative scale, thus the duration in the respective placebo group was given the value of 100%. The difference between zinc and placebo groups thus directly indicates the effect of zinc lozenges in percentages. See Additional file 2 for the extraction of data and for the calculation of the relative mean and SD values for the duration of symptoms, and Additional file 3 for the raw data and the estimates for individual studies.

Figure 3 shows the effect of zinc acetate lozenges on systemic symptoms. There was no heterogeneity between the three studies on the effects of zinc lozenges on fever, muscle ache or headache (I^2^ = 0% for each symptom). The pooled effects indicate that zinc shortens the duration of muscle ache by 54%, whereas the 13% shorter duration of headaches and the 35% shorter duration of fevers were not significantly different between the zinc acetate and placebo groups.

**Figure 4:**
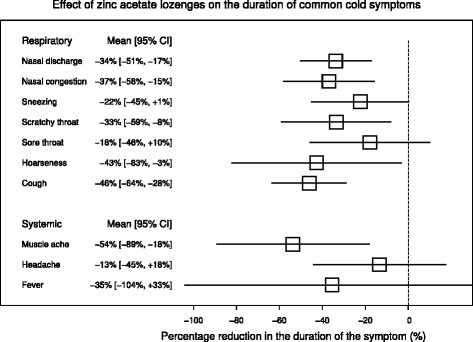
**The effect of high dose zinc acetate lozenges on the duration of common cold symptoms.** The pooled estimates and their 95% CIs are shown in this figure. The horizontal lines indicate the 95% CI for the effect and the squares in the middle of the horizontal lines indicate the point estimates of the effect on the particular respiratory and systemic symptoms. See Additional files 2 and 3 for the calculations.

Figure 4 summarizes the effects of zinc acetate lozenges on the respiratory and systemic symptoms. There is no heterogeneity in the effect of zinc on the seven respiratory symptoms with I^2^ = 0% (Additional file 3, p. 4), indicating that the variation in the zinc effect on the seven respiratory symptoms might be just due to random variation. The pooled effects on sneezing and sore throat were not significant; however, the 95% CIs for both symptoms are wide and they are not inconsistent with the effects on other symptoms. There was strong evidence that zinc acetate lozenges shortened the duration of muscle ache. The 95% CIs for headache and fever are wide.

**Figure 5:**
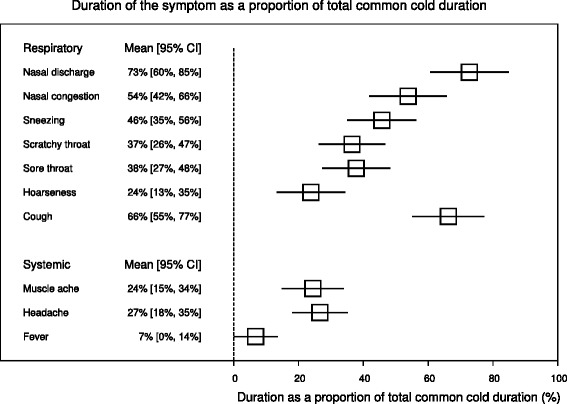
**The duration of the specific symptom as a proportion of the total common cold duration (set as 100%) of the placebo groups.** The left side of the figure shows the duration of the symptom as a proportion of the total common cold duration, and the right side shows the same information as a forest plot. On the scale of this figure, 100% corresponds to the total duration of the common cold of the placebo groups. The duration of symptoms was calculated as follows. First, the duration of the specific symptom was calculated as the percentage of the total common cold duration for the placebo group (5.1 days in Petrus et al. [21], 8.1 days in Prasad et al. (2000) [22], and 7.1 days in Prasad et al. (2008) [23]). Then the relative duration of each specific symptom was pooled using the RevMan program. There was no heterogeneity between the three trials in the relative duration of untreated nasal discharge, sneezing, sore throat, hoarseness, muscle pain, headache, and fever with P > 0.05 for the test of heterogeneity. The relative durations of untreated nasal congestion (I^2^ = 85%), scratchy throat (I^2^ = 74%), and cough (I^2^ = 68%) were significantly different in the three trials. See Additional files 2 and 4 for the calculations.

There is substantial variation between the mean durations of individual symptoms. For example, in the placebo group of the Prasad et al. (2008) trial, muscle ache lasted for a mean of 2.0 days, but cough lasted for 5.1 days; whereas the total duration of colds was 7.1 days (Table 3). Therefore the percentage effect of zinc on the specific symptoms should be considered together with the duration of untreated symptoms. Figure 5 shows the duration of symptoms so that they are normalized by the total duration of the colds in the three trials. For example, in the three trials, mean nasal discharge lasted for 73% (95% CI 60% to 85%) of the total common cold duration.

**Table 3:** **The effect of zinc acetate lozenges on common cold symptoms in the Prasad et al. (2008) study** [23]

	**Treatment group**			
	**Zinc**	**Placebo**	**Effect of zinc**	**Effect of zinc**
	**(Days; mean)**	**(Days; mean)**	**(Days; mean, 95% CI)**	**(%; mean, 95% CI)**
Total duration	4.00	7.12	−3.12 (−2.48, −3.76)	−43% (−34%, −53%)
Nasal discharge	3.00	4.56	−1.56 (−0.22, −2.90)	−34% (−4%, −64%)
Cough	2.16	5.08	−2.92 (−1.58, −4.26)	−57% (−30%, −84%)
Muscle ache	0.80	2.00	−1.20 (−0.20, −2.20)	−60% (−9%, −110%)

The symptom durations were longest for nasal discharge, nasal congestion and cough so that each of them lasted for more than half of the total duration of the common cold (Figure 5). Thus, time-wise, the effect of zinc lozenges on these symptoms is more clinically important than the effect on shorter-lived symptoms. These three longest-lasting respiratory symptoms were all shortened by 34% or more, with particularly small P-values (Figures 2 and 4). The systemic symptoms lasted for only a quarter or less of the total common cold duration (Figure 5). Therefore, the 54% reduction on muscle ache corresponded to a shorter time period free of the symptom than the 34% to 46% effects on the longer-lasting respiratory symptoms.

Calculating the relative effect of zinc lozenges is the best approach to pool the results of the three trials since it adjusts for baseline variations in symptom duration. On the other hand, the absolute effect of zinc on the days saved of illness is also important as it has practical relevance for patients. As an illustration of the effect of zinc on both the days saved and the percentage shortening of symptoms, Table 3 shows the findings of the most recent study by Prasad et al. (2008). Only those outcomes on which zinc had a significant influence are shown, since the point estimates of the effect are useful only for them. Muscle ache was shortened by 1.2 days, which is less than half of the 2.9 day effect on cough, yet the relative effect is the same for both. This is explained by the substantially longer mean duration of cough, so that the same relative effect corresponds to more actual days saved from coughing. Nasal discharge was shortened by 1.5 days, which is half of the effect on cough. In this comparison the non-treated duration is essentially the same, but the relative effect is smaller for nasal discharge.

## Discussion

Given that zinc lozenges are slowly dissolved in the pharyngeal region, and that the effects of zinc seem to be local, there might be variation in the effects of zinc lozenges on common cold symptoms in different anatomical regions. Contrary to such reasoning, the effects of zinc lozenges on the nasal symptoms were not significantly different compared with the symptoms that originated in the lower anatomical regions. Furthermore, there was no heterogeneity between the effect of zinc on the seven respiratory symptoms with I^2^ = 0%. This indicates that the lack of significance in the effects on sneezing and sore throat might simply be false negative findings, as their confidence intervals substantially overlap with all the other symptoms (Figure 4). Nevertheless, the substantial overlap between the 95% CIs and the I^2^ = 0% do not necessarily indicate that the size of the effect of zinc lozenges on all the seven respiratory symptoms is the same. Instead, the sizes of the studies were probably insufficient to reveal modest differences in the effects on the seven respiratory symptoms.

The anatomical origin of the three nasal symptoms, sore and scratchy throat, and hoarseness (laryngitis) is evident. However, cough has a more ambiguous anatomical origin. It can originate from different anatomical regions with different pathologies, and thus is not similarly informative of the possible locations of zinc lozenge effects [28,29].

The common cold is usually caused by respiratory viruses that have over 100 serotypes [30]. The distribution of viruses varies over time and geography and therefore the common cold episodes in different controlled trials have different aetiologies. In addition, variations in outcome definitions also generate variation between trials and the interventions are also not identical. For these reasons, variation between trials investigating the effects of common cold treatments is to be expected. Nevertheless, only for sore throat and cough was there substantial heterogeneity in the effect of zinc acetate lozenges over the three included studies with I^2^ above 60% (Figure 2).

The most common cause for the common cold is rhinovirus, and usually the most bothersome symptom of rhinovirus infections is nasal discharge [31]. Therefore, the 34% reduction in the duration of nasal discharge, with no heterogeneity between the three trials, is a particularly important effect (Figure 4). The common cold is the most common cause of acute cough [29] and therefore the estimated 46% reduction in the duration of cough is also very important (Figure 4).

In the three trials analyzed, the systemic symptoms of colds were much shorter than the respiratory symptoms. Muscle ache and headache lasted for a quarter and fever lasted for less than a tenth of the total common cold duration (Figure 5). Only muscle ache was shortened significantly by zinc. Nevertheless, it seems probable that the three studies did not have sufficient statistical power to estimate the effect of zinc acetate lozenges on headache and fever since the pooled 95% CIs were wide for both symptoms.

All included studies were randomized double-blind, placebo-controlled trials, and the level of drop outs was none or few. Therefore, the risk of bias in the three included trials is low (Table 2). All of the three trials reported seven of the most important respiratory symptoms (Figure 2). Zinc did not have a significant effect on 13 of 21 respiratory symptoms across the 3 studies, yet the data on the 13 symptoms were still reported. Thus, all 7 symptoms were reported for each of the 3 studies. Consequently, there is no basis to assume that reporting bias might confound our analysis of these specific common cold symptoms.

Two of the included trials [22,23] only enrolled participants who had had cold symptoms for less than 24 hours. The third study did not describe the duration of illness before zinc administration, but most of the participants started treatment on the first day of enrolment in the study [21]. Hence it is possible that the observed benefit of zinc in the two studies [22,23] might not have occurred had the delay between the onset of the common cold and the initiation of zinc treatment been longer than one day.

In the USA, the recommended dietary zinc intake is 11 mg/day for men and 8 mg/day for women [32]. Thus, the 80 to 92 mg/day doses used in the zinc acetate lozenge trials are substantially higher than the recommended daily intakes. However, in several clinical trials zinc has been administered to patients at a dose of 150 mg/day for months [33-37]. A decrease in copper levels and haematological changes have been reported as adverse effects of long-term high dose zinc administration, but those changes were completely reversed with the cessation of zinc intake [38-42]. Thus, given that 150 mg/day of zinc administration for months does not cause permanent harm, it seems plausible that the use of about 80 mg/day of zinc for up to two weeks in the form of zinc acetate lozenges is unlikely to cause serious adverse effects.

In some zinc lozenge trials the lozenges caused short-term adverse effects, such as bad taste, but the bad taste can be explained by the specific lozenge composition and does not necessarily reflect the effects of zinc ions themselves [9,10]. None of the high dose zinc acetate lozenge trials reported bad taste to be a problem and there was no substantial difference between the zinc and placebo groups in the recorded adverse effects, and only a few drop-outs occurred. Furthermore, if a common cold patient suffers from acute adverse effects such as bad taste, the patient can simply stop taking the zinc acetate lozenges.

Evidence-based medicine focuses primarily on the effect of interventions on clinically relevant outcomes in controlled trials, which is also the approach of this meta-analysis. Nevertheless, the potential biological mechanisms are also of interest. Although the mechanism of zinc in the alleviation of colds is not known, possible mechanisms have been proposed. In laboratory studies zinc inhibited the replication of respiratory viruses and enhanced the effect of interferons [10,43,44]. Non-immune mechanisms have also been proposed to explain the effect of zinc lozenges on the common cold [10,45,46]. However, the lack of well-formulated mechanistic explanations should not hamper the implications of these three randomized trials with clinically relevant outcomes.

Although these three trials that used high dose zinc acetate lozenges show that an appropriate lozenge composition can substantially shorten the duration of various common cold symptoms, many zinc lozenges on the US market either have too low a dose of zinc or contain ingredients that tightly bind to zinc ions, such as citric acid [10]. Therefore, the full benefit seen in the three high dose zinc acetate trials may not be easy to actualize until high dose zinc acetate lozenges are more widely available on the market.

## Conclusions

We found no evidence that the effect of high dose zinc acetate lozenges varies between the respiratory symptoms originating from different anatomic regions. Given that there were only few and minor adverse effects in the three randomized trials, zinc acetate lozenges may be a useful treatment option for the common cold. Since two of the included studies started zinc treatment within 24 hours of the onset of the common cold, the strongest evidence of benefit is for such rapid initiation of zinc administration. More research is needed to find optimal lozenge compositions and treatment strategies.

### Availability of supporting data

Supporting data is available as additional files.

## Additional files

Additional file 1:
**Flow diagram: search and selection of the included trials.**
Additional file 2:
**Extraction of data and transformation to percentage scale.**
Additional file 3:
**RevMan program outputs showing raw data for the calculation of the effect of zinc acetate.**
Additional file 4:
**RevMan program outputs showing raw data for the calculation of the proportion of the duration of specific symptoms of the total common cold duration.**

## References

[1] Eby GA, Davis DR, Halcomb WW (1984). Reduction in duration of common cold by zinc gluconate lozenges in a double-blind study. Antimicrob Agents Chemother.

[2] Godfrey JC (1988). Zinc for the common cold. Antimicrob Agents Chemother.

[3] Eby GA (1988). Stability constants of zinc complexes affect common cold treatment results. Antimicrob Agents Chemother.

[4] Martin RB (1988). pH as a variable in free zinc ion concentration from zinc-containing lozenges. Antimicrob Agents Chemother.

[5] Zarembo JE, Godfrey JC, Godfrey NJ (1992). Zinc(II) in saliva: determination of concentrations produced by different formulations of zinc gluconate lozenges containing common excipients. J Pharm Sci.

[6] Eby GA (1997). Zinc ion availability: the determinant of efficacy in zinc lozenge treatment of common colds. J Antimicrob Chemother.

[7] Bakar NKA, Taylor DM, Williams DR (1999). The chemical speciation of zinc in human saliva: possible correlation with reduction of the symptoms of the common cold produced by zinc gluconate-containing lozenges. Chem Speciat Bioavailab.

[8] Eby GA (2001). Elimination of efficacy by additives in zinc acetate lozenges for common colds. Clin Infect Dis.

[9] Eby GA (2004). Zinc lozenges: cold cure or candy? Solution chemistry determinations. Biosci Rep.

[10] Eby GA (2010). Zinc lozenges as cure for the common cold. Med Hypotheses.

[11] Hemilä H (2011). Zinc lozenges may shorten the duration of colds: a systematic review. Open Respir Med J.

[12] Singh M, Das RR. Zinc for the common cold. Cochrane Database Syst Rev 2013, CD001364. http://www.ncbi.nlm.nih.gov/pubmed/2377570510.1002/14651858.CD001364.pub4 10.1002/14651858.CD001364.pub4 23775705

[13] Science M, Johnstone J, Roth DE, Guyatt G, Loeb M (2012). Zinc for the treatment of the common cold: a systematic review and meta-analysis of randomized controlled trials. CMAJ.

[14] Hemilä H. Errors in the Cochrane review (2011) on zinc for the common cold. PubMed Commons (2014 Dec 6), http://1.usa.gov/1vWPRnt

[15] Hemilä H. Concerns about unattributed copying of text and data, and about numerous other problems in the Cochrane review “Zinc for the Common Cold” by Singh M, Das RR (2013). 2015. http://hdl.handle.net/10138/153180.

[16] Hemilä H. Zinc acetate lozenges may shorten common cold duration by up to 40%. CMAJ eLetter May 28, 2012. http://www.cmaj.ca/content/184/10/E551/reply#cmaj_el_706238 Available at: http://hdl.handle.net/10138/40083 10.1586/ers.12.30 22788939

[17] Hirt M, Nobel S, Barron E (2000). Zinc nasal gel for the treatment of common cold symptoms: a double-blind, placebo-controlled trial. Ear Nose Throat J.

[18] Mossad SB (2003). Effect of zincum gluconicum nasal gel on the duration and symptom severity of the common cold in otherwise healthy adults. QJM.

[19] Jafek BW, Linschoten MR, Murrow BW (2004). Anosmia after intranasal zinc gluconate use. Am J Rhinol.

[20] Alexander TH, Davidson TM (2006). Intranasal zinc and anosmia: the zinc-induced anosmia syndrome. Laryngoscope.

[21] Petrus EJ, Lawson KA, Bucci LR, Blum K (1998). Randomized, double-masked, placebo-controlled clinical study of the effectiveness of zinc acetate lozenges on common cold symptoms in allergy-tested subjects. Curr Ther Res.

[22] Prasad AS, Fitzgerald JT, Bao B, Beck FW, Chandrasekar PH (2000). Duration of symptoms and plasma cytokine levels in patients with the common cold treated with zinc acetate. A randomized, double-blind, placebo-controlled trial. Ann Intern Med.

[23] Prasad AS, Beck FW, Bao B, Snell D, Fitzgerald JT (2008). Duration and severity of symptoms and levels of plasma interleukin-1 receptor antagonist, soluble tumor necrosis factor receptor, and adhesion molecules in patients with common cold treated with zinc acetate. J Infect Dis.

[24] Prisma statement. http://www.prisma-statement.org/

[25] Review Manager (RevMan) [Computer program]. Version 5.3. Copenhagen: The Nordic Cochrane Centre, The Cochrane Collaboration, 2014. http://ims.cochrane.org/revman

[26] Higgins JPT, Thompson SG, Deeks JJ, Altman DG (2003). Measuring inconsistency in meta-analysis. BMJ.

[27] Gnuplot [Computer program]. http://www.gnuplot.info

[28] Pratter MR (2006). Cough and the common cold: ACCP evidence-based clinical practice guidelines. Chest.

[29] Dicpinigaitis PV, Morice AH, Birring SS, McGarvey L, Smith JA, Canning BJ (2014). Antitussive drugs: past, present, and future. Pharmacol Rev.

[30] Weber O, Eccles R, Weber O (2009). The role of viruses in the etiology and pathogenesis of common cold. Common Cold.

[31] Arruda E, Pitkäranta A, Witek TJ, Doyle CA, Hayden FG (1997). Frequency and natural history of rhinovirus infections in adults during autumn. J Clin Microbiol.

[32] National Research Council (2001). Dietary Reference Intakes for Vitamin A, Vitamin K, Arsenic, Boron, Chromium, Copper, Iodine, Iron, Manganese, Molybdenum, Nickel, Silicon, Vanadium, and Zinc.

[33] Pories WJ, Henzel JH, Rob CG, Strain WH (1967). Acceleration of healing with zinc sulfate. Ann Surg.

[34] Hallböök T, Laner E (1972). Serum-zinc and healing of venous leg ulcers. Lancet.

[35] Barcia PJ (1970). Lack of acceleration of healing with zinc sulfate. Ann Surg.

[36] Simkin PA (1976). Oral zinc sulphate in rheumatoid arthritis. Lancet.

[37] Bamford JT, Gessert CE, Haller IV, Kruger K, Johnson BP (2012). Randomized, double-blind trial of 220 mg zinc sulfate twice daily in the treatment of rosacea. Int J Dermatol.

[38] Samman S, Roberts DC (1987). The effect of zinc supplements on plasma zinc and copper levels and the reported symptoms in healthy volunteers. Med J Aust.

[39] Prasad AS, Brewer GJ, Schoomaker EB, Rabbani P (1978). Hypocupremia induced by zinc therapy in adults. JAMA.

[40] Hoffman HN, Phyliky RL, Fleming CR (1988). Zinc-induced copper deficiency. Gastroenterology.

[41] Forman WB, Sheehan D, Cappelli S, Coffman B (1990). Zinc abuse: an unsuspected cause of sideroblastic anemia. West J Med.

[42] Fiske DN, McCoy HE, Kitchens CS (1994). Zinc-induced sideroblastic anemia: report of a case, review of the literature, and description of the hematologic syndrome. Am J Hematol.

[43] Korant BD, Butterworth BE (1976). Inhibition by zinc of rhinovirus protein cleavage: interaction of zinc with capsid polypeptides. J Virol.

[44] Berg K, Bolt G, Andersen H, Owen TC (2001). Zinc potentiates the antiviral action of human IFN-alpha tenfold. J Interferon Cytokine Res.

[45] Novick SG, Godfrey JC, Godfrey NJ, Wilder HR (1996). How does zinc modify the common cold? Clinical observations and implications regarding mechanisms of action. Med Hypotheses.

[46] Eby G (2012). The mouth-nose biologically closed electric circuit in zinc lozenge therapy of common colds as explanation of rapid therapeutic action. Expert Rev Respir Med.

