# Supplementary material for: The effectiveness of high dose zinc acetate lozenges on various common cold symptoms: a meta-analysis
Source: BMC Fam Pract. 2015 Feb 25;16:24. doi: 10.1186/s12875-015-0237-6 (PMC4359576; doi:10.1186/s12875-015-0237-6)
Supplement: Additional file 1: — Flow diagram: search and selection of the included trials. [file 12875_2015_237_MOESM1_ESM.pdf]

Hemilä (2011) (ref 11):
Zinc lozenges and the common cold, placebo-controlled trials

13 trials

Singh & Das (2013) (ref 12):
Zinc for treating and preventing the common cold, randomized trials

18 trials

Science et al. (2012) (ref 13):
Zinc for treatment of the common cold, randomized trials

17 trials

Restriction to placebo-controlled trials on high dose zinc lozenges (>75 mg/day zinc) and the common cold

3 trials

3 trials

3 trials

Removal of duplicates

3 trials

New search of Pubmed
with the free search terms "zinc" and "lozenge$"
(Jan 2, 2015)
Overall 28 records identified.

No new trials found

3 placebo-controlled trials with zinc lozenges in doses >75 mg/day of zinc were included in the meta-analysis: refs. 21-23
