# Supplementary material for: The effectiveness of high dose zinc acetate lozenges on various common cold symptoms: a meta-analysis
Source: BMC Fam Pract. 2015 Feb 25;16:24. doi: 10.1186/s12875-015-0237-6 (PMC4359576; doi:10.1186/s12875-015-0237-6)

Additional File 3 to:

The effectiveness of high dose zinc acetate lozenges on various common cold symptoms: a meta-analysis

Harri Hemilä and Elizabeth Chalker

Submitted to: BMC Family Practice
http://www.biomedcentral.com/bmcfampract

This file describes the intermediate calculations with RevMan to estimate the effect of high dose zinc acetate lozenges on the duration of various common cold symptoms.

Harri Hemilä
Department of Public Health
University of Helsinki
Helsinki, Finland
harri.hemila@helsinki.fi
http://www.mv.helsinki.fi/home/hemila/

Contents

Page

2 A version of Fig. 2 to show the raw data and estimates of effect on respiratory symptoms in the included studies. The figure was generated using RevMan.

3 A version of Fig. 3 to show the raw data and estimates of effect on systemic symptoms in the included studies. The figure was generated using RevMan.

4 RevMan Figure showing the pooled effects of zinc acetate lozenges on respiratory and systemic symptom durations. This figure was redrawn with Gnuplot for the report as Fig. 4.

	Zinc			Placebo				Mean Difference
	Mean	SD	Total	Mean	SD	Total	Weight	IV, Fixed, 95% CI
**1.2.1 Nasal discharge**								
Petrus 1998	61	53	52	100	92	49	8.0%	-39.00 [-68.51, -9.49]
Prasad 2000	71	34	25	100	60	23	8.9%	-29.00 [-56.91, -1.09]
Prasad 2008	66	36	25	100	66	25	8.0%	-34.00 [-63.47, -4.53]
**Subtotal (95% CI)**			**102**			**97**	**24.9%**	**-33.81 [-50.52, -17.11]**
Heterogeneity: Chi² = 0.23, df = 2 (P = 0.89); I² = 0%								
Test for overall effect: Z = 3.97 (P < 0.0001)								
**1.2.2 Nasal congestion**								
Petrus 1998	52	51	52	100	93	49	8.0%	-48.00 [-77.50, -18.50]
Prasad 2000	70	52	25	100	77	23	4.9%	-30.00 [-67.49, 7.49]
Prasad 2008	86	79	25	100	113	25	2.4%	-14.00 [-68.05, 40.05]
**Subtotal (95% CI)**			**102**			**97**	**15.3%**	**-36.90 [-58.21, -15.60]**
Heterogeneity: Chi² = 1.36, df = 2 (P = 0.51); I² = 0%								
Test for overall effect: Z = 3.39 (P = 0.0007)								
**1.2.3 Sneezing**								
Petrus 1998	75	82	52	100	130	49	3.8%	-25.00 [-67.68, 17.68]
Prasad 2000	62	44	25	100	76	23	5.5%	-38.00 [-73.53, -2.47]
Prasad 2008	102	62	25	100	87	25	4.0%	2.00 [-39.88, 43.88]
**Subtotal (95% CI)**			**102**			**97**	**13.3%**	**-22.33 [-45.21, 0.54]**
Heterogeneity: Chi² = 2.06, df = 2 (P = 0.36); I² = 3%								
Test for overall effect: Z = 1.91 (P = 0.06)								
**1.2.4 Scratchy throat**								
Petrus 1998	47	71	52	100	98	49	6.2%	-53.00 [-86.55, -19.45]
Prasad 2000	100	78	25	100	116	23	2.2%	0.00 [-56.41, 56.41]
Prasad 2008	88	80	25	100	120	25	2.2%	-12.00 [-68.53, 44.53]
**Subtotal (95% CI)**			**102**			**97**	**10.5%**	**-33.55 [-59.23, -7.86]**
Heterogeneity: Chi² = 3.21, df = 2 (P = 0.20); I² = 38%								
Test for overall effect: Z = 2.56 (P = 0.01)								
**1.2.5 Sore throat**								
Petrus 1998	162	176	52	100	156	49	1.7%	62.00 [-2.78, 126.78]
Prasad 2000	67	61	25	100	97	23	3.2%	-33.00 [-79.30, 13.30]
Prasad 2008	61	57	25	100	91	25	3.9%	-39.00 [-81.09, 3.09]
**Subtotal (95% CI)**			**102**			**97**	**8.8%**	**-17.83 [-45.90, 10.24]**
Heterogeneity: Chi² = 7.22, df = 2 (P = 0.03); I² = 72%								
Test for overall effect: Z = 1.25 (P = 0.21)								
**1.2.6 Hoarseness**								
Petrus 1998	43	97	52	100	197	49	1.9%	-57.00 [-118.14, 4.14]
Prasad 2000	167	202	25	100	231	23	0.5%	67.00 [-56.22, 190.22]
Prasad 2008	46	66	25	100	132	25	2.1%	-54.00 [-111.85, 3.85]
**Subtotal (95% CI)**			**102**			**97**	**4.4%**	**-42.66 [-82.43, -2.89]**
Heterogeneity: Chi² = 3.40, df = 2 (P = 0.18); I² = 41%								
Test for overall effect: Z = 2.10 (P = 0.04)								
**1.2.7 Cough**								
Petrus 1998	110	136	52	100	121	49	2.8%	10.00 [-40.14, 60.14]
Prasad 2000	49	39	25	100	52	23	10.2%	-51.00 [-77.18, -24.82]
Prasad 2008	43	34	25	100	59	25	9.8%	-57.00 [-83.69, -30.31]
**Subtotal (95% CI)**			**102**			**97**	**22.7%**	**-46.14 [-63.65, -28.63]**
Heterogeneity: Chi² = 5.58, df = 2 (P = 0.06); I² = 64%								
Test for overall effect: Z = 5.16 (P < 0.00001)								

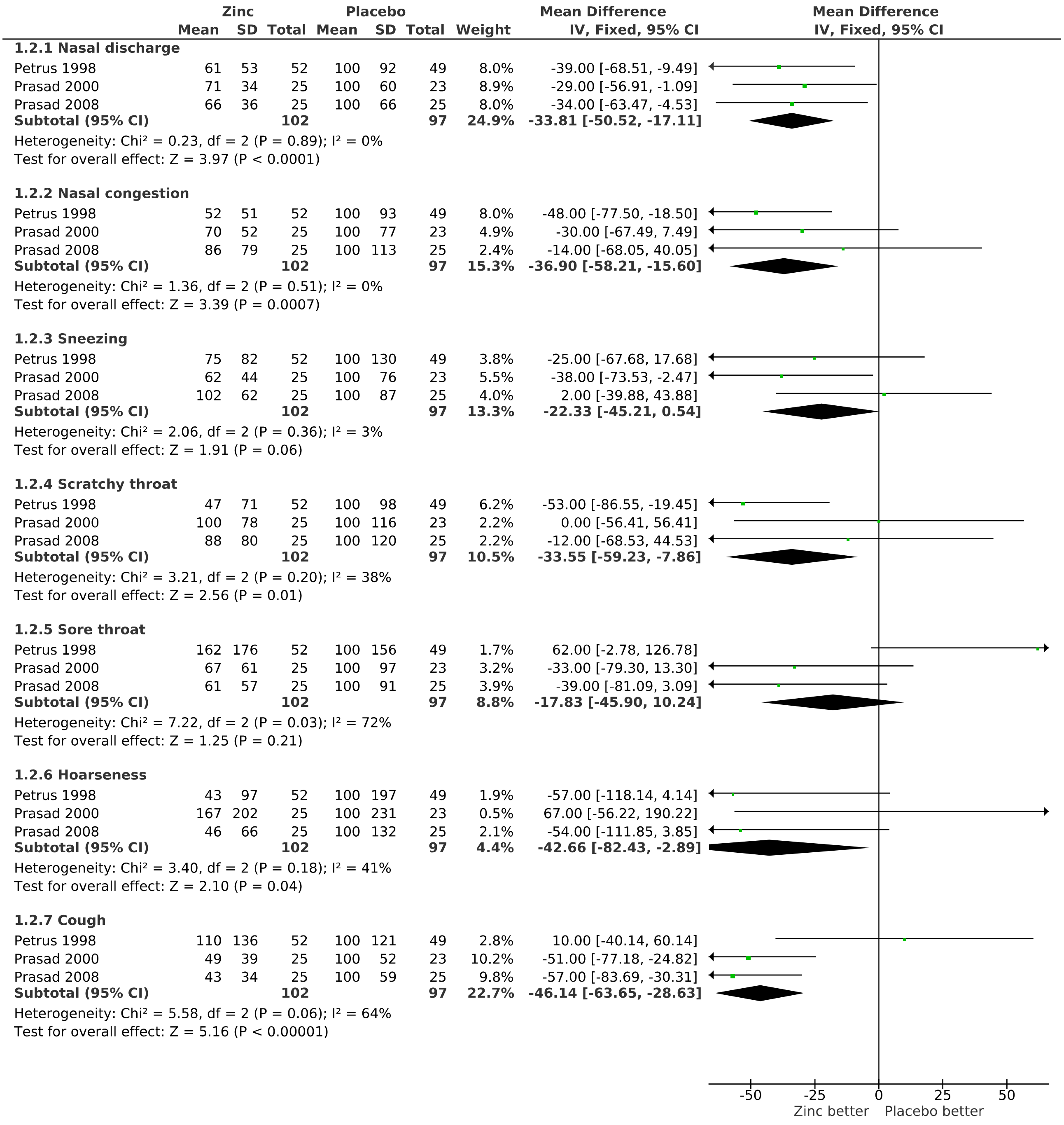

Study or Subgroup	Zinc Mean	Zinc SD	Zinc Total	Placebo Mean	Placebo SD	Placebo Total	Weight	Mean Difference IV, Fixed, 95% CI
**1.3.1 Muscle ache**								
Petrus 1998	35	120	52	100	182	49	13.4%	-65.00 [-125.50, -4.50]
Prasad 2000	93	129	25	100	186	23	5.9%	-7.00 [-98.30, 84.30]
Prasad 2008	40	61	25	100	113	25	19.4%	-60.00 [-110.34, -9.66]
**Subtotal (95% CI)**			**102**			**97**	**38.6%**	**-53.66 [-89.29, -18.03]**
Heterogeneity: Chi² = 1.20, df = 2 (P = 0.55); I² = 0%								
Test for overall effect: Z = 2.95 (P = 0.003)								
**1.3.2 Headache**								
Petrus 1998	95	125	52	100	142	49	17.9%	-5.00 [-57.30, 47.30]
Prasad 2000	83	86	25	100	97	23	18.1%	-17.00 [-69.04, 35.04]
Prasad 2008	81	89	25	100	116	25	14.9%	-19.00 [-76.31, 38.31]
**Subtotal (95% CI)**			**102**			**97**	**51.0%**	**-13.36 [-44.38, 17.65]**
Heterogeneity: Chi² = 0.15, df = 2 (P = 0.93); I² = 0%								
Test for overall effect: Z = 0.84 (P = 0.40)								
**1.3.3 Fever**								
Petrus 1998	65	263	52	100	408	49	2.7%	-35.00 [-169.76, 99.76]
Prasad 2000	200	484	25	100	349	23	0.9%	100.00 [-137.36, 337.36]
Prasad 2008	47	121	25	100	179	25	6.8%	-53.00 [-137.69, 31.69]
**Subtotal (95% CI)**			**102**			**97**	**10.4%**	**-35.53 [-104.18, 33.11]**
Heterogeneity: Chi² = 1.42, df = 2 (P = 0.49); I² = 0%								
Test for overall effect: Z = 1.01 (P = 0.31)								

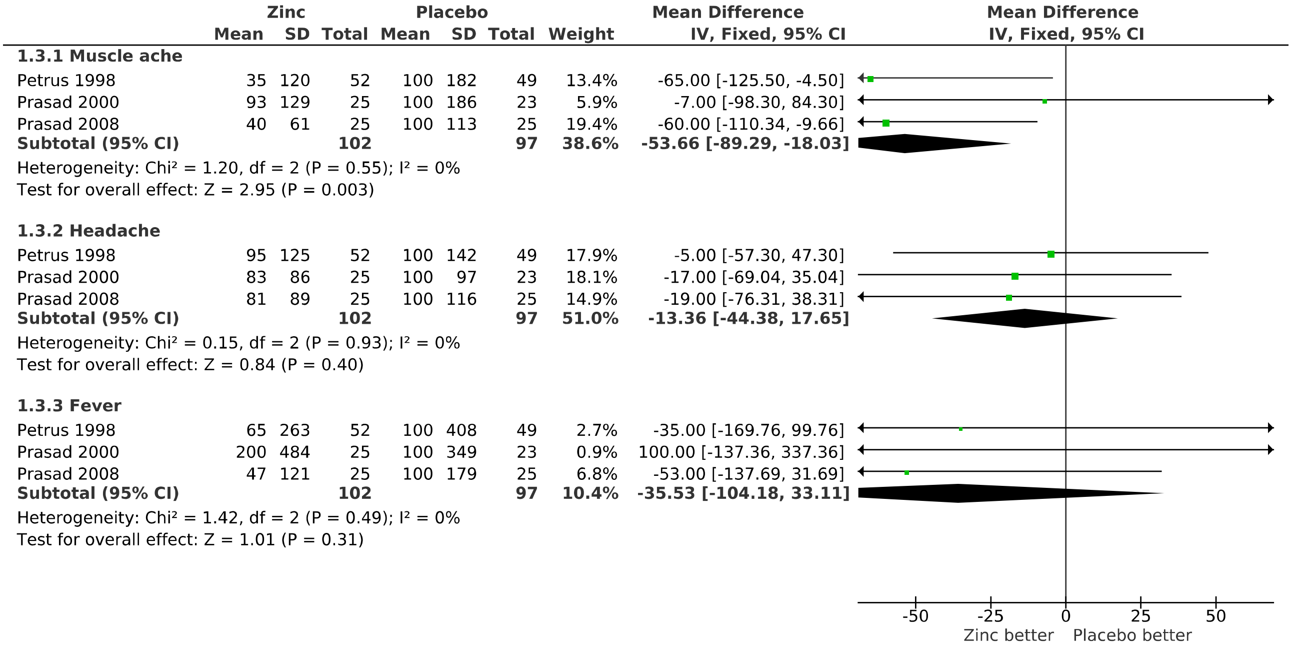

	Mean Difference	SE	Weight	Mean Difference IV, Fixed, 95% CI
**2.1.1 Respiratory**				
Nasal discharge	-33.81	8.52	21.8%	-33.81 [-50.51, -17.11]
Nasal congestion	-36.9	10.87	13.4%	-36.90 [-58.20, -15.60]
Sneezing	-22.33	11.67	11.6%	-22.33 [-45.20, 0.54]
Scratchy thoroat	-33.55	13.1	9.2%	-33.55 [-59.23, -7.87]
Sore throat	-17.83	14.32	7.7%	-17.83 [-45.90, 10.24]
Hoarseness	-42.66	20.29	3.9%	-42.66 [-82.43, -2.89]
Cough	-46.14	8.93	19.9%	-46.14 [-63.64, -28.64]
**Subtotal (95% CI)**			**87.6%**	**-34.51 [-42.85, -26.17]**
Heterogeneity: $Chi^2 = 4.36$, df = 6 (P = 0.63); $I^2 = 0\%$				
Test for overall effect: Z = 8.11 (P < 0.00001)				
**2.1.2 Systemic**				
Muscle ache	-53.66	18.18	4.8%	-53.66 [-89.29, -18.03]
Headache	-13.36	15.82	6.3%	-13.36 [-44.37, 17.65]
Fever	-35.53	35.02	1.3%	-35.53 [-104.17, 33.11]
**Subtotal (95% CI)**			**12.4%**	**-31.23 [-53.37, -9.09]**
Heterogeneity: $Chi^2 = 2.81$, df = 2 (P = 0.24); $I^2 = 29\%$				
Test for overall effect: Z = 2.76 (P = 0.006)				

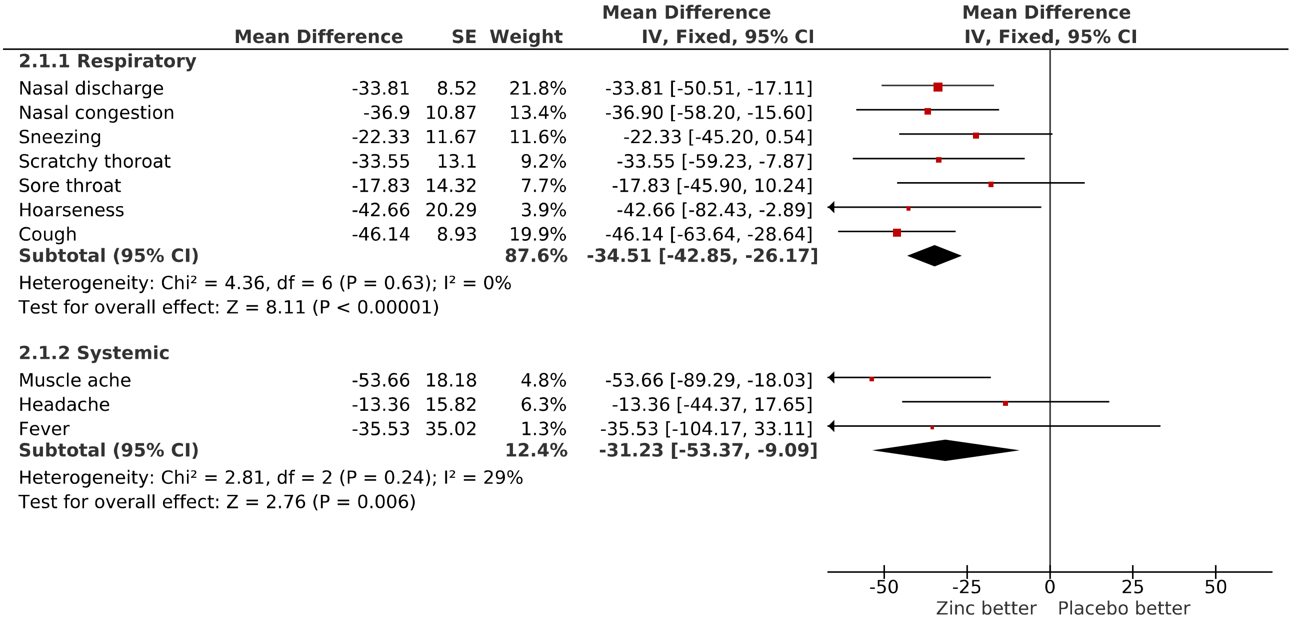

Supplement: Additional file 3: — RevMan program outputs showing raw data for the calculation of the effect of zinc acetate. [file 12875_2015_237_MOESM3_ESM.pdf]
