# Supplementary material for: The effectiveness of high dose zinc acetate lozenges on various common cold symptoms: a meta-analysis
Source: BMC Fam Pract. 2015 Feb 25;16:24. doi: 10.1186/s12875-015-0237-6 (PMC4359576; doi:10.1186/s12875-015-0237-6)

Additional File 4 to:

The effectiveness of high dose zinc acetate lozenges on various common cold symptoms: a meta-analysis

Harri Hemilä and Elizabeth Chalker

Submitted to: BMC Family Practice
http://www.biomedcentral.com/bmcfampract

This file describes the intermediate calculations with RevMan to calculate the proportion of cold symptoms of the total common cold duration

Harri Hemilä
Department of Public Health
University of Helsinki
Helsinki, Finland
harri.hemila@helsinki.fi
http://www.mv.helsinki.fi/home/hemila/

Contents

Page

2 RevMan Figure showing the pooled proportion of respiratory symptoms of the total common cold durations in the placebo groups.

3 RevMan Figure showing the pooled proportion of systemic symptoms of the total common cold durations in the placebo groups.

4 RevMan Figure showing the pooled durations of the respiratory and systemic symptoms in the placebo groups. This figure was redrawn with Gnuplot for the report as Fig. 5.

	Symptom			Total cold				Mean Difference
	Mean	SD	Total	Mean	SD	Total	Weight	IV, Fixed, 95% CI
**3.1.1 Nasal discharge**								
Petrus 1998	0	92	49	100	57.9	49	1.9%	-100.00 [-130.44, -69.56]
Prasad 2000	28	43	23	100	22.2	23	4.4%	-72.00 [-91.78, -52.22]
Prasad 2008	36	42	25	100	17.7	25	5.4%	-64.00 [-81.87, -46.13]
**Subtotal (95% CI)**			**97**			**97**	**11.7%**	**-72.76 [-84.92, -60.61]**
Heterogeneity: Chi² = 4.01, df = 2 (P = 0.13); I² = 50%								
Test for overall effect: Z = 11.73 (P < 0.00001)								
**3.1.2 Nasal congestion**								
Petrus 1998	-2	94	49	100	57.9	49	1.8%	-102.00 [-132.91, -71.09]
Prasad 2000	42	44	23	100	22.2	23	4.3%	-58.00 [-78.14, -37.86]
Prasad 2008	64	40	25	100	17.7	25	5.9%	-36.00 [-53.15, -18.85]
**Subtotal (95% CI)**			**97**			**97**	**11.9%**	**-53.84 [-65.86, -41.81]**
Heterogeneity: Chi² = 13.65, df = 2 (P = 0.001); I² = 85%								
Test for overall effect: Z = 8.77 (P < 0.00001)								
**3.1.3 Sneezing**								
Petrus 1998	39	79	49	100	57.9	49	2.3%	-61.00 [-88.42, -33.58]
Prasad 2000	46	42	23	100	22.2	23	4.6%	-54.00 [-73.41, -34.59]
Prasad 2008	63	32	25	100	17.7	25	8.4%	-37.00 [-51.33, -22.67]
**Subtotal (95% CI)**			**97**			**97**	**15.3%**	**-45.70 [-56.33, -35.07]**
Heterogeneity: Chi² = 3.31, df = 2 (P = 0.19); I² = 40%								
Test for overall effect: Z = 8.43 (P < 0.00001)								
**3.1.4 Scratchy throat**								
Petrus 1998	33	66	49	100	57.9	49	2.9%	-67.00 [-91.58, -42.42]
Prasad 2000	65	40	23	100	22.2	23	4.9%	-35.00 [-53.70, -16.30]
Prasad 2008	73	32	25	100	17.7	25	8.4%	-27.00 [-41.33, -12.67]
**Subtotal (95% CI)**			**97**			**97**	**16.2%**	**-36.49 [-46.82, -26.17]**
Heterogeneity: Chi² = 7.63, df = 2 (P = 0.02); I² = 74%								
Test for overall effect: Z = 6.93 (P < 0.00001)								
**3.1.5 Sore throat**								
Petrus 1998	72	43	49	100	57.9	49	4.2%	-28.00 [-48.19, -7.81]
Prasad 2000	63	36	23	100	22.2	23	5.8%	-37.00 [-54.29, -19.71]
Prasad 2008	54	41	25	100	17.7	25	5.6%	-46.00 [-63.51, -28.49]
**Subtotal (95% CI)**			**97**			**97**	**15.6%**	**-37.81 [-48.31, -27.30]**
Heterogeneity: Chi² = 1.76, df = 2 (P = 0.42); I² = 0%								
Test for overall effect: Z = 7.05 (P < 0.00001)								
**3.1.6 Hoarseness**								
Petrus 1998	72	55	49	100	57.9	49	3.5%	-28.00 [-50.36, -5.64]
Prasad 2000	85	34	23	100	22.2	23	6.3%	-15.00 [-31.59, 1.59]
Prasad 2008	69	41	25	100	17.7	25	5.6%	-31.00 [-48.51, -13.49]
**Subtotal (95% CI)**			**97**			**97**	**15.3%**	**-23.79 [-34.40, -13.19]**
Heterogeneity: Chi² = 1.87, df = 2 (P = 0.39); I² = 0%								
Test for overall effect: Z = 4.40 (P < 0.0001)								
**3.1.7 Cough**								
Petrus 1998	57	52	49	100	57.9	49	3.6%	-43.00 [-64.79, -21.21]
Prasad 2000	22	40	23	100	22.2	23	4.9%	-78.00 [-96.70, -59.30]
Prasad 2008	29	42	25	100	17.7	25	5.4%	-71.00 [-88.87, -53.13]
**Subtotal (95% CI)**			**97**			**97**	**14.0%**	**-66.19 [-77.30, -55.08]**
Heterogeneity: Chi² = 6.16, df = 2 (P = 0.05); I² = 68%								
Test for overall effect: Z = 11.68 (P < 0.00001)								

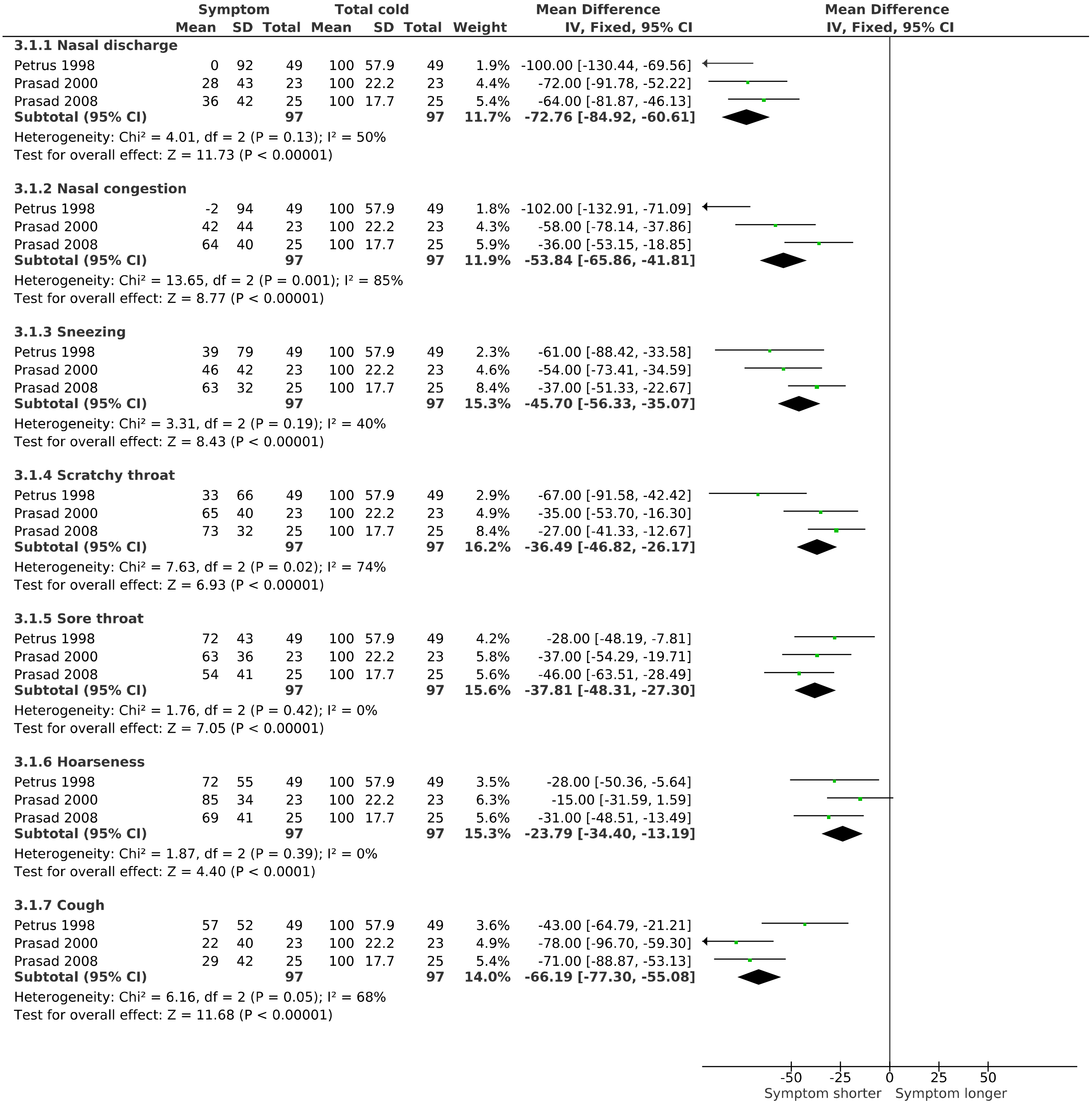

	Symptom			Total cold				Mean Difference
	Mean	SD	Total	Mean	SD	Total	Weight	IV, Fixed, 95% CI
**3.2.1 Muscle ache**								
Petrus 1998	75	45	49	100	57.9	49	5.3%	-25.00 [-45.53, -4.47]
Prasad 2000	81	34	23	100	22.2	23	8.2%	-19.00 [-35.59, -2.41]
Prasad 2008	72	32	25	100	17.7	25	10.9%	-28.00 [-42.33, -13.67]
**Subtotal (95% CI)**			**97**			**97**	**24.4%**	**-24.34 [-33.93, -14.75]**
Heterogeneity: Chi² = 0.65, df = 2 (P = 0.72); I² = 0%								
Test for overall effect: Z = 4.97 (P < 0.00001)								
**3.2.2 Headache**								
Petrus 1998	60	57	49	100	57.9	49	4.3%	-40.00 [-62.75, -17.25]
Prasad 2000	70	29	23	100	22.2	23	10.1%	-30.00 [-44.93, -15.07]
Prasad 2008	79	24	25	100	17.7	25	16.4%	-21.00 [-32.69, -9.31]
**Subtotal (95% CI)**			**97**			**97**	**30.9%**	**-26.61 [-35.14, -18.08]**
Heterogeneity: Chi² = 2.41, df = 2 (P = 0.30); I² = 17%								
Test for overall effect: Z = 6.11 (P < 0.00001)								
**3.2.3 Fever**								
Petrus 1998	96	16	49	100	57.9	49	7.9%	-4.00 [-20.82, 12.82]
Prasad 2000	97.53	9	23	100	22.2	23	23.4%	-2.47 [-12.26, 7.32]
Prasad 2008	84	28	25	100	17.7	25	13.3%	-16.00 [-28.98, -3.02]
**Subtotal (95% CI)**			**97**			**97**	**44.7%**	**-6.77 [-13.86, 0.31]**
Heterogeneity: Chi² = 2.79, df = 2 (P = 0.25); I² = 28%								
Test for overall effect: Z = 1.87 (P = 0.06)								

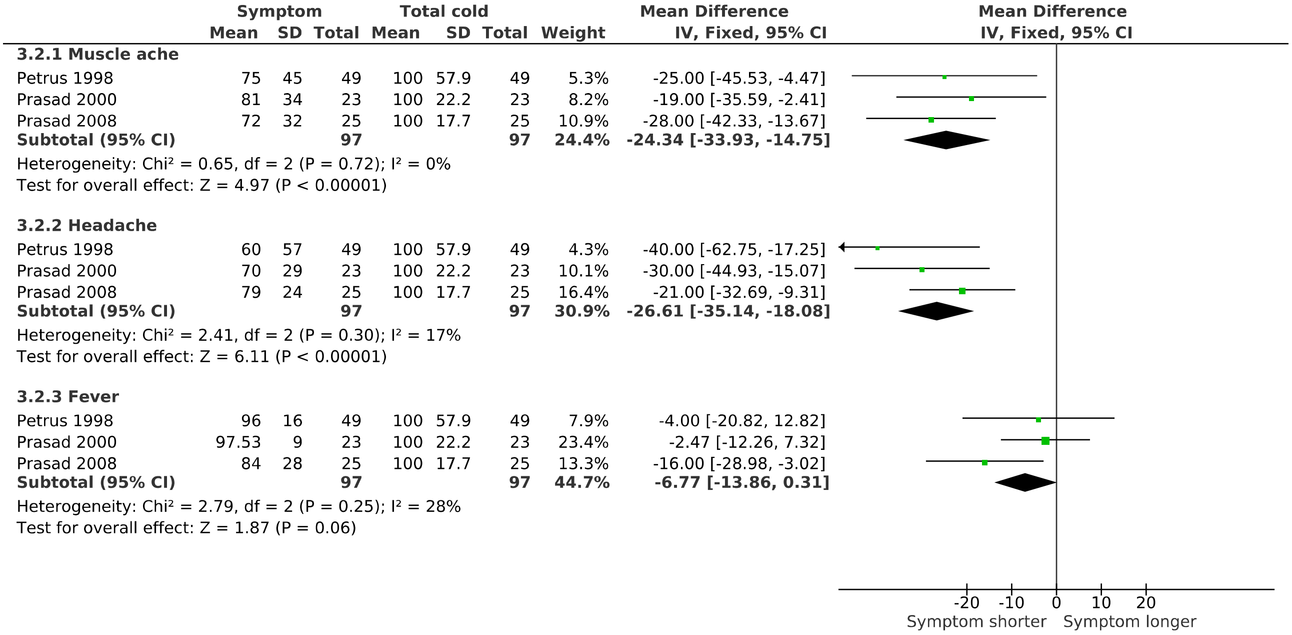

	Mean Difference	SE	Weight	Mean Difference IV, Fixed, 95% CI
**4.1.1 Respiratory**				
Nasal discharge	72.76	6.2	6.5%	72.76 [60.61, 84.91]
Nasal congestion	53.84	6.14	6.7%	53.84 [41.81, 65.87]
Sneezing	45.7	5.42	8.5%	45.70 [35.08, 56.32]
Scratchy thoroat	36.49	5.27	9.0%	36.49 [26.16, 46.82]
Sore throat	37.81	5.36	8.7%	37.81 [27.30, 48.32]
Hoarseness	23.79	5.41	8.6%	23.79 [13.19, 34.39]
Cough	66.19	5.92	7.2%	66.19 [54.59, 77.79]
**Subtotal (95% CI)**			**55.3%**	**46.38 [42.20, 50.56]**
Heterogeneity: Chi^2 = 54.31, df = 6 ($P < 0.00001$); I^2 = 89%				
Test for overall effect: $Z = 21.76$ ($P < 0.00001$)				
**4.1.2 Systemic**				
Muscle ache	24.34	4.89	10.5%	24.34 [14.76, 33.92]
Headache	26.61	4.35	13.3%	26.61 [18.08, 35.14]
Fever	6.77	3.46	21.0%	6.77 [-0.01, 13.55]
**Subtotal (95% CI)**			**44.7%**	**16.78 [12.13, 21.42]**
Heterogeneity: Chi^2 = 15.87, df = 2 ($P = 0.0004$); I^2 = 87%				
Test for overall effect: $Z = 7.08$ ($P < 0.00001$)				

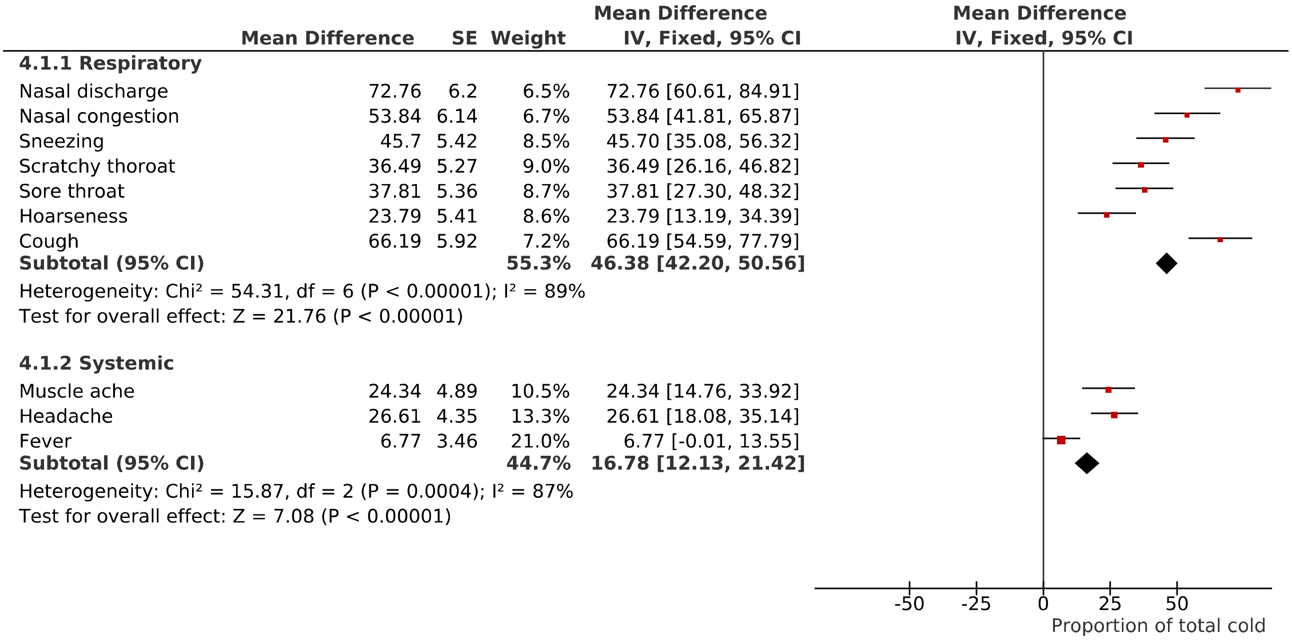

Supplement: Additional file 4: — RevMan program outputs showing raw data for the calculation of the proportion of the duration of specific symptoms of the total common cold duration. [file 12875_2015_237_MOESM4_ESM.pdf]
